# Efficiency of genomic prediction across two *Eucalyptus nitens* seed orchards with different selection histories

**DOI:** 10.1038/s41437-018-0119-5

**Published:** 2018-07-06

**Authors:** Mari Suontama, Jaroslav Klápště, Emily Telfer, Natalie Graham, Toby Stovold, Charlie Low, Russell McKinley, Heidi Dungey

**Affiliations:** Scion (The New Zealand Forest Research Institute Ltd.), 49 Sala Street, Rotorua, 3046 New Zealand

**Keywords:** Plant breeding, Genomics

## Abstract

Genomic selection is expected to enhance the genetic improvement of forest tree species by providing more accurate estimates of breeding values through marker-based relationship matrices compared with pedigree-based methodologies. When adequately robust genomic prediction models are available, an additional increase in genetic gains can be made possible with the shortening of the breeding cycle through elimination of the progeny testing phase and early selection of parental candidates. The potential of genomic selection was investigated in an advanced *Eucalyptus nitens* breeding population focused on improvement for solid wood production. A high-density SNP chip (EUChip60K) was used to genotype 691 individuals in the breeding population, which represented two seed orchards with different selection histories. Phenotypic records for growth and form traits at age six, and for wood quality traits at age seven were available to build genomic prediction models using GBLUP, which were compared to the traditional pedigree-based alternative using BLUP. GBLUP demonstrated that breeding value accuracy would be improved and substantial increases in genetic gains towards solid wood production would be achieved. Cross-validation within and across two different seed orchards indicated that genomic predictions would likely benefit in terms of higher predictive accuracy from increasing the size of the training data sets through higher relatedness and better utilization of LD

## Introduction

Shining gum, *Eucalyptus nitens* (Deane & Maiden), is the most important commercial eucalypt species in New Zealand, with an advanced breeding programme moving towards its fourth generation (Klápště et al. 2017). The New Zealand *E. nitens* breeding programme is an open-pollinated (OP) breeding population, therefore, some inaccuracy in breeding values is expected due to unknown paternal contribution and plausible pedigree errors. Precisely recorded pedigrees and known ancestors of the breeding population individuals are prerequisites for accurate genetic evaluation and consequently, efficient breeding programme management and boosted genetic gain in forest tree species.

Genomic selection was proposed as a tool to predict individual breeding values on the basis of information from high-density genetic marker panels through multiple regression models (Meuwissen et al. 2001). Development of next generation sequencing technologies such as genotyping by sequencing (Elshire et al. 2011) or exome capture (Neves et al. 2013) has allowed implementation of genomic technologies in species with missing reference genomes such as forest trees (Resende et al. 2012a; Resende et al. 2012b; Ratcliffe et al. 2015; El-Dien et al. 2015; Beaulieu et al. 2014a).

High-density genotyping of a sufficiently large sub-sample of the target breeding population offers a tool for more precise selection of breeding candidates (Resende et al. 2012b) by fitting marker-based relationship matrices instead of documented pedigrees (Nejati-Javaremi et al. 1997; Van Raden 2008). The advantage of marker-based relationship matrices is that gaps are filled in pairwise relatedness in forest tree pedigrees, which leads to an increase in the accuracy of genetic parameters and more precise selections of breeding candidates (Zapata-Valenzuela et al. 2013; Isik et al. 2016; Müller et al. 2017; Tan et al. 2017). The genomic prediction model generally captures: (1) shared genealogy, (2) co-segregation and (3) linkage disequilibrium (LD) between markers and quantitative trait loci (QTL). The contribution of each of these is affected by the genetic architecture of a trait, genomic marker density and marker distribution and effective population size (Habier et al. 2013). The accuracy of genomic prediction also depends on a trait’s heritability, training population size and the effective number of chromosomal segments, defined as the function of a trait’s genetic architecture (distribution of QTLs) and decay of LD along the chromosome (Hayes et al. 2009). The level of relatedness between training and validation population is also an important factor affecting the accuracy of genomic breeding values (Habier et al. 2010; Scutari et al. 2016).

*Eucalyptus nitens* has been grown predominantly for pulp wood production with short rotations in Southland, the southernmost region of New Zealand and the major *E. nitens* plantation area. Breeding objectives have been for improved growth and form (Wilcox 1980; King and Wilcox 1988), whilst wood quality traits have not been the focus of breeding until recently, with an increasing interest in the use of *E. nitens* for higher value solid wood products. Similar trends in breeding of eucalypt species for wood quality have been reported in previous studies (Raymond 2002; Kube 2005; Kube and Raymond 2005; Grattapaglia and Kirst 2008; Hamilton et al. 2009). Earlier research efforts on implementing molecular genetics in breeding of *E. nitens* have included QTL trait locus and candidate gene approaches for growth and vegetation traits; and wood quality where a number of candidate genes for wood quality and QTLs for growth and vegetative propagation traits were identified (Thumma et al. 2010a, 2010b). Initiation of genomic estimated breeding values were proposed in the latest breeding plan of *E. nitens* in New Zealand, with a focus on solid wood production. Implementation of genomic estimated breeding values is expected to accelerate the rate of genetic progress for the breeding objective traits because of greater accuracy of genetic evaluation and the shorter generation interval than in traditional breeding (Resende et al. 2012; Resende et al. 2012b).

The objective of this study was to investigate the improvement in accuracy of genetic evaluation for growth, form and wood quality traits when using marker-based breeding values with GBLUP methodology compared to pedigree-based BLUP in an advanced *E. nitens* population. Firstly, the aim was to estimate heritability and accuracy of pedigree and marker-based breeding values within and across the progeny of two seed orchards. Secondly, the aim was to compare predictive accuracy of BLUP and GBLUP estimated breeding values within and across the seed orchards. Finally, implications of the results for the breeding programme and the next steps in the research will be discussed.

## Materials and methods

### Materials

The *E. nitens* population used in this study was established as an OP progeny test of families from two independent seed orchards, Waiouru and Tinkers. The number of families in the progeny test originating from the Waiouru seed orchard was 90, and the number of families in the progeny test from the Tinkers seed orchard was 25. In the current study, 51 and 100% of the families were represented from the Waiouru and Tinkers seed orchards, respectively. Outline of the breeding programme history is described by Klápště et al. (2017).

The third generation progeny trial used in this study was located in the South Island of New Zealand. The total number of individuals in the trial was 3600, with a subsample of 691 individuals representing 72 families with 1–24 (average of 9.6) individuals per family used for genomic prediction analysis for which genomic information and phenotypic trait records were available. The Waiouru seed orchard was represented by 431 genotyped individuals with effective population size of 133.2, while the Tinkers seed orchard was represented by 236 genotyped individuals with effective population size of 64.7. The effective population size (*N*_*E*_) was estimated in terms of status number as following $N_S = \frac{1}{{2\theta }}$, where *Ɵ* is group co-ancestry of individuals (Lindgren et al. 1996). The remaining genotyped individuals represented Australian Tree Seed Centre orchard (ATSC) as control trees in the progeny test. Trees in this progeny test were assessed at age six for tree height, diameter at breast height (DBH) and stem straightness. These same trees were assessed at age seven for the following wood quality traits: wood density, wood stiffness, wood shrinkage and growth strain. Methodology to measure wood quality traits is described in detail by Klápště et al. (2017).

**Fig. 1:**
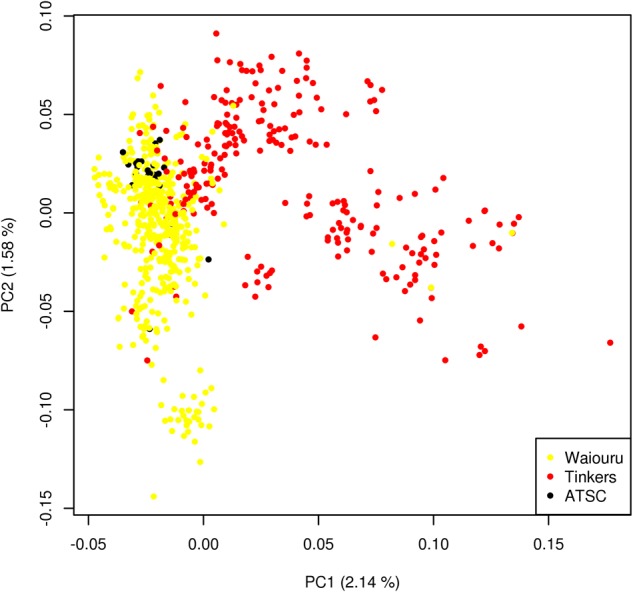
Spectral decomposition of the genetic marker-based relationship matrix

**Fig. 2:**
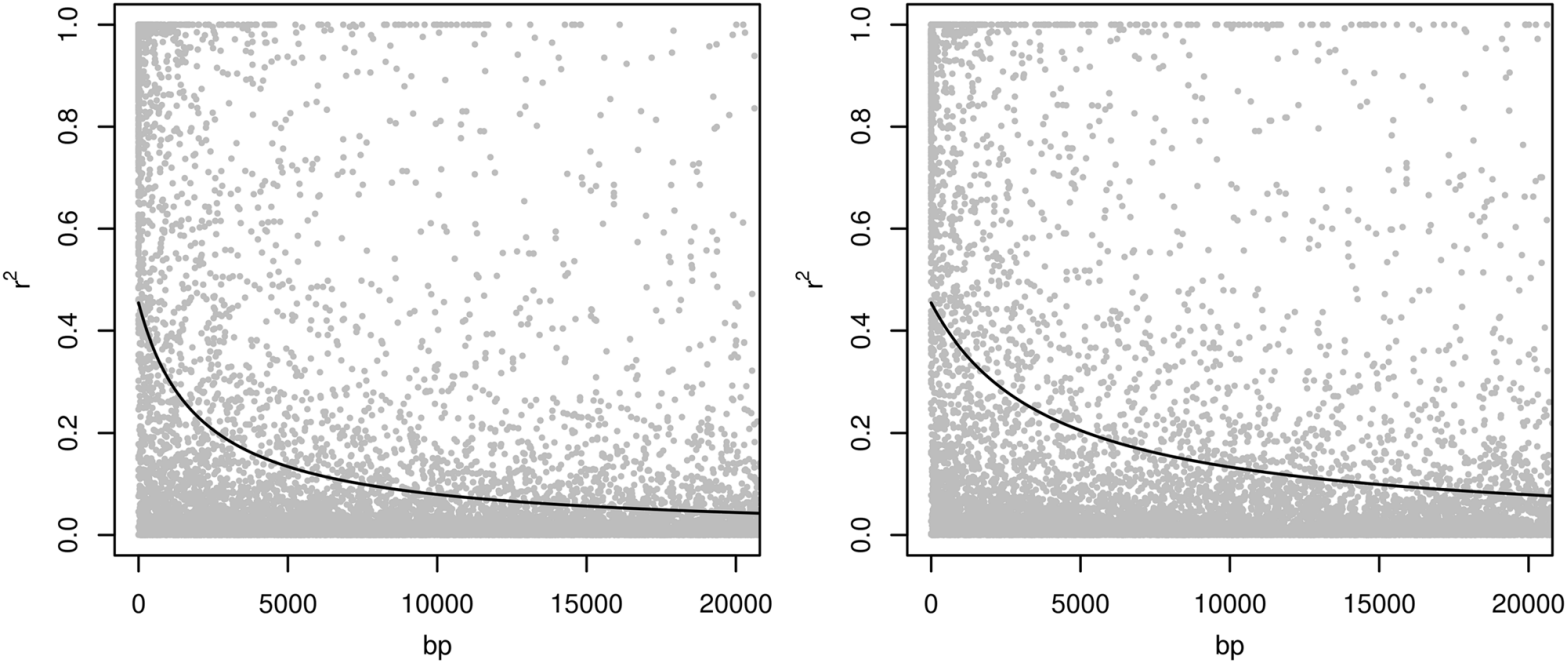
Linkage disequilibrium decay with physical distance in base pairs within Waiouru (left plot) and Tinkers (right plot) samples

Genomic data was generated by extracting DNA from the leaf tissue of 691 individuals from the progeny trial using the commercially available NucleoSpin® 96 Plant II kit (Machery-Nagel, Dϋren, Germany) (Telfer et al. 2013) and sent to GeneSeek, Inc. (a Neogen company, Lincoln, NE, USA) for genotyping. Genotyping was undertaken using the Illumina Infinium EUChip60K SNP chip (Silva‐Junior et al. 2015) with SNP calling performed on the basis of multi-taxa and *Maidenaria* section reference. Both call algorithms produced a similar number of SNPs (58,307 vs. 58,323). The marker data were filtered for genTrain score > 0.5, GenCall > 0.15, minor allele frequency > 0.01, SNP call rate > 0.6 and pairwise LD in terms of a composite estimate (*r*^2^ < 0.9), with 12,236 SNPs selected to train genomic prediction models. The missing data were imputed through expectation-maximisation algorithm implemented in “rrBLUP” package (Endelman 2011). The spectral decomposition of the realized relationship matrix showed a clear segregation of each seed orchard population with Australian Tree Seed Centre (ATSC) families in between. This reflects the differences in the genetic background of these populations due to different selection strategies (Fig. 1). LD decreased to 0.2 within 3 kb in Waiouru and within 5 kb in Tinkers (Fig. 2).

### Methods

The genetic parameters were estimated using linear mixed models implemented in the ASReml-R package (Butler et al. 2009). Two models using either a pedigree or marker-based relationship matrix were investigated and compared. A pedigree-based model (BLUP) was used as follows:

1 $${\boldsymbol{y}} = {\mathbf{X}}{\boldsymbol{\beta }} + {\mathbf{Z}}_1{\boldsymbol{u}} + {\mathbf{Z}}_2{\boldsymbol{r}} + {\mathbf{Z}}_3{\boldsymbol{r}}\left( {\boldsymbol{s}} \right) + {\boldsymbol{e}}$$

where ***y*** is a vector of measurements, ***β*** is a vector of fixed terms such as intercept and seed source, ***u*** is a vector of additive genetic effects (breeding values) following $var\left( u \right)\sim N(0,{\sigma _a^2}{\mathrm{{A}}})$, where $\sigma _a^2$ is additive genetic variance and **A** is the average numerator relationship matrix (Wright, 1922), ***r*** is a vector of random replication effects following $var\left( r \right)\sim N\left( {0,\sigma _r^2{{\mathbf{I}}}} \right)$ where $\sigma _r^2$ is replication variance and **I** is the identity matrix, ***r(s)*** is set nested within replication following $var\left( {r\left( s \right)} \right)\sim N\left( {0,\sigma _{r\left( S \right)}^2{\mathbf{I}}} \right)$ where $\sigma _{r(s)}^2$ is set nested within replication variance. Set is an incomplete block effect within a replicate where the equal number of families were allocated to each set within a replicate. A vector of random residuals is **e** following $var\left( e \right)\sim N\left( {0,\sigma _e^2{\mathbf{I}}} \right)$, where $\sigma _e^2$ is residual variance, **X** and **Z**_**1**_**, Z**_**2**_ and **Z**_**3**_ are incidence matrices assigning fixed and random effects to measurements in vector ***y*** (Klápště et al. 2017). The model accommodating a marker-based relationship matrix (GBLUP) was performed using the previous Eq. (1), but the average numerator relationship matrix **A** was substituted by a marker-based relationship matrix **G** which was estimated as follows:

2 $${{\mathbf{G}}} = \frac{{{\mathbf{ZZ}}\prime }}{{\mathrm{tr}\left[ {{\mathbf{ZZ}}\prime } \right]/n}}$$

where **Z** is **M** – **P**, **M** is the marker matrix with genotypes coded 0, 1 and 2 for alternative allele homozygote, heterozygote and reference allele homozygote, respectively, and **P** is a vector of twice the reference allele frequency, tr[**ZZ**’] is a trace of the matrix defined in nominator and *n* is the number of markers (Forni et al. 2011). Heritability represents the proportion of a trait’s variance explained by genetic factors and can provide inference about the potential efficiency of any genetic improvement (Falconer and Mackay 1996). Narrow-sense heritability was estimated as:

3 $$\widehat h^2 = \frac{{\widehat \sigma _a^2}}{{\widehat \sigma _a^2 + \widehat \sigma _e^2}}$$

where $\sigma _a^2$ is additive genetic variance and $\sigma _e^2$ is residual variance. The accuracy of breeding values represents correlation of their estimates obtained from the model [1] with their true breeding values which are commonly unknown. The theoretical accuracy of breeding values is estimated using the following formula:

4 $$r = \sqrt {1 - \frac{{\mathrm{PEV}}}{{G_{ii}\sigma _a^2}}}$$

where PEV is prediction error variance (Mrode 2014) and *G*_*ii*_ is the diagonal element of the marker-based relationship matrix for the i^th^ individual and is substituted by *A*_*ii*_ in the pedigree-based scenario.

A 10-fold cross-validation performed through 30 replications was used as independent evaluation, and was performed at an individual level, and within, between, and across seed orchards. The resulting predictive accuracies indicate the efficiency of the marker-based model as a prediction tool for breeding values based solely on marker information. Such a scenario reflects the main advantages of genomic selection in breeding programmes: the elimination of the testing phase (establishment of progeny trials) from breeding cycles and selections based only on genetic markers. It was estimated as follows:

5 $$r_p = cor\left( {\mathrm{EBV}}\,{\mathrm{GEBV}} \right)$$

where EBV is the vector of breeding values estimated by the pedigree-based model and GEBV is the vector of breeding values predicted in cross-validation using the marker-based model.

The full efficiency of genomic selection was investigated by comparing genetic gains per generation using pedigree-based estimated breeding values (BLUP) with genetic gains using genomic marker-based estimated breeding values (GBLUP). Both of these individual based estimated genetic gains included the availability of phenotypic records for all traits. The mean value of BLUP and GBLUP breeding values of the 20% of selected individuals was the measurement for the estimated genetic gain.

## Results

### Estimates of heritability and accuracy of breeding values

Pedigree-based analysis showed low to moderate within seed orchard heritabilities in both seed orchards (Table 1). Pedigree-based estimated heritabilities were higher at Tinkers than at Waiouru for radial wood shrinkage, tangential air-dry wood shrinkage and wood stiffness at 1.4–3 m traits, but lower for all other traits at Tinkers. Marker-based within seed orchard heritabilities was also low to moderate, and generally higher at Waiouru than at Tinkers, except for tangential air-dry wood shrinkage and growth strain (Table 1).

**Table 1:** Estimates of narrow sense heritability (*h*^2^) with their standard errors (se) and breeding value accuracies (*r*) within seed orchards

Trait	Waiouru						Tinkers					
	BLUP			GBLUP			BLUP			GBLUP		
	*h* ^2^	se	*r*	*h* ^2^	se	*r*	*h* ^2^	se	*r*	*h* ^2^	se	*r*
Radial air-dry wood shrinkage (%)	0.26	0.110	0.55	0.35	0.074	0.70	0.52	0.141	0.73	0.22	0.062	0.60
Radial reconditioned wood shrinkage (%)	0.25	0.110	0.54	0.33	0.073	0.68	0.58	0.146	0.77	0.24	0.063	0.62
Tangential air-dry wood shrinkage (%)	0.22	0.106	0.51	0.20	0.066	0.58	0.31	0.117	0.59	0.37	0.072	0.71
Tangential reconditioned wood shrinkage (%)	0.51	0.139	0.73	0.50	0.067	0.78	0.43	0.132	0.68	0.39	0.070	0.72
Wood density (kg/m^3^)	0.54	0.141	0.74	0.52	0.068	0.79	0.34	0.117	0.62	0.42	0.061	0.74
Diameter at breast height (mm)	0.00	0.00	0.00	0.14	0.062	0.51	0.29	0.112	0.57	0.03	0.039	0.29
Height (m)	0.00	0.00	0.00	0.14	0.062	0.51	0.29	0.112	0.57	0.03	0.039	0.29
Stem straightness (score)	0.41	0.130	0.66	0.19	0.063	0.57	0.11	0.089	0.37	0.12	0.059	0.48
Wood stiffness 1.4–3.0 m log (km/s)	0.28	0.112	0.56	0.34	0.072	0.69	0.35	0.120	0.62	0.24	0.060	0.62
Wood stiffness 3.0–6.0 m log (km/s)	0.19	0.100	0.48	0.30	0.071	0.66	0.15	0.099	0.44	0.18	0.057	0.56
Growth strain 1.4–3.0 m log (mm)	NA	NA	NA	0.21	0.064	0.59	NA	NA	NA	0.25	0.061	0.62
Growth strain 3.0–6.0 m log (mm)	0.25	0.111	0.53	0.23	0.066	0.60	0.24	0.111	0.52	0.30	0.066	0.66

*NA* Log likelihood not converged

Across seed orchard heritability estimates were generally higher using marker-based breeding values than pedigree-based with only a few exceptions (Table 2). Reconditioned radial wood shrinkage, DBH, height and stem straightness indicated lower heritability estimates using marker-based models than when pedigree-based models were used. The highest heritabilities were estimated for wood shrinkage and wood density traits. The lowest heritability estimates were obtained for DBH and height and were relatively similar with both pedigree and marker-based methods.

**Table 2:** Estimates of narrow sense heritability (*h*^2^) with their standard errors (se) and breeding value accuracies (*r*) and estimated genetic gains per generation (ΔG_BLUP_, ΔG_GBLUP_) across seed orchards

Trait	BLUP				GBLUP			
	*h* ^2^	se	*r*	ΔG_BLUP_	*h* ^2^	se	*r*	ΔG_GBLUP_
Radial air-dry wood shrinkage (%)	0.31	0.116	0.59	0.25	0.33	0.073	0.68	0.29
Radial reconditioned wood shrinkage (%)	0.41	0.128	0.66	0.16	0.31	0.071	0.67	0.15
Tangential air-dry wood shrinkage (%)	0.37	0.123	0.63	0.56	0.50	0.073	0.77	0.79
Tangential reconditioned wood shrinkage (%)	0.44	0.131	0.68	0.28	0.49	0.070	0.77	0.34
Wood density (kg/m^3^)	0.44	0.130	0.68	17.66	0.46	0.067	0.76	20.29
Diameter at breast height (mm)	0.09	0.085	0.34	2.35	0.08	0.052	0.42	2.87
Height (m)	0.09	0.085	0.34	0.12	0.08	0.052	0.42	0.15
Stem straightness (score)	0.28	0.115	0.57	0.47	0.19	0.064	0.57	0.39
Wood stiffness 1.4–3.0 m log (km/s)	0.24	0.108	0.52	0.07	0.29	0.070	0.65	0.09
Wood stiffness 3.0–6.0 m log (km/s)	0.12	0.094	0.39	0.04	0.17	0.063	0.55	0.07
Growth strain 1.4–3.0 m log (mm)	NA	NA	NA	NA	0.23	0.065	0.60	2.28
Growth strain 3.0–6.0 m log (mm)	0.16	0.101	0.44	1.46	0.24	0.068	0.60	2.69

*NA* Log likelihood not converged

Marker-based breeding values had consistently higher accuracies than pedigree-based breeding values. Generally, accuracies of breeding values were higher for both seed orchards when using marker-based rather than pedigree-based estimated breeding values. However, there were some inconsistencies, with the Tinkers seed orchard showing lower genomic breeding value accuracies for radial wood shrinkage, DBH and height than for the same traits using pedigree-based models, reflecting the pattern in heritability.

### Cross-validation

The cross-validation analysis showed no, or very low, predictive accuracy for breeding values for one seed orchard when based on a model trained in an alternative seed orchard, using pedigree-based or marker-based models (Table 3). Within seed orchard predictive accuracy, however, improved considerably for both seed orchards and for the majority of traits when using marker-based models. Pedigree-based models resulted in lower predictive accuracy for most of the traits within Waiouru seed orchard compared with Tinkers seed orchard, but this trend was not so obvious with marker-based models. Across seed orchards, predictive accuracies were higher for marker-based analysis than for pedigree-based predictions for the majority of traits.

**Table 3:** Predictive accuracy from cross validation scenarios on individual BLUP and GBLUP breeding values within the Waiouru (W) and Tinkers (T) seed orchards and across the both seed orchards (WT)

Cross validation scenario BLUP → Training Validation						Cross validation scenario GBLUP→Training Validation				
Trait	W → T	W → W	T → W	T → T	WT → WT	W → T	W → W	T → W	T → T	WT → WT
Radial air-dry wood shrinkage (%)	−0.01	0.22	−0.03	0.35	0.29	0.03	0.33	0.00	0.36	0.34
Radial reconditioned wood shrinkage (%)	−0.07	0.12	−0.12	0.26	0.19	0.03	0.21	0.04	0.31	0.26
Tangential air-dry wood shrinkage (%)	−0.09	0.13	0.03	0.20	0.16	0.08	0.19	0.08	0.37	0.26
Tangential reconditioned wood shrinkage (%)	−0.07	0.28	−0.02	0.18	0.27	0.07	0.38	0.07	0.38	0.38
Wood density (kg/m^3^)	−0.03	0.36	−0.01	0.27	0.32	0.02	0.49	0.03	0.52	0.46
Diameter at breast height (mm)	0.15	0.13	0.10	0.43	0.28	−0.03	0.26	0.04	0.13	0.22
Height (m)	0.19	0.13	0.10	0.42	0.27	−0.03	0.26	0.04	0.15	0.21
Stem straightness (score)	−0.09	0.04	−0.04	−0.01	0.02	−0.01	0.05	−0.04	−0.04	0.02
Wood stiffness 1.4–3.0 m log (km/s)	0.10	0.25	0.02	0.31	0.28	0.04	0.33	−0.02	0.38	0.33
Wood stiffness 3.0–6.0 m log (km/s)	−0.06	0.28	0.02	0.16	0.21	0.03	0.32	−0.04	0.24	0.25
Growth strain 1.4–3.0 m log (mm)	0.29	0.22	0.13	0.27	0.24	−0.01	0.30	−0.01	0.36	0.30
Growth strain 3.0–6.0 m log (mm)	0.23	0.27	0.14	0.19	0.24	−0.05	0.35	−0.07	0.40	0.35

### Estimates of genetic gains

Generally estimated absolute genetic gains per generation were as expected higher when using GBLUP breeding values rather than BLUP breeding values (Table 2). The estimated genetic gains reflected the magnitude of heritability for the traits. The largest difference in genetic gains between BLUP and GBLUP was for growth strain in the upper part of the log, which is one of the most important trait in selection for solid wood production of this species. Stem straightness was the only trait that had a significantly lower genetic gain when using GBLUP than BLUP.

## Discussion

The benefits of using information from genomic markers in the genetic evaluation were apparent in the current study, where both within seed orchard and across seed orchard estimations supported the use of GBLUP over BLUP predictions. The reduced number of SNPs after filtering still provided sufficient genomic information to perform efficient marker-based predictions, which improved breeding value accuracies when compared with pedigree-based evaluations. A gain in accuracy of breeding values would likely be even more substantial when increasing the size of training population by additional genotyping. Grattapaglia and Resende (2011) estimated that the impact of training population size on the accuracy of genomic breeding values would increase up to a sample size of 2000. However, the effect of the training population size on accuracy of breeding values also depends on genomic marker density and the number of QTL controlling the trait (Grattapaglia and Resende 2011). The size and composition of the training sets and the number of SNPs were found more important factors in genomic prediction, than statistical methodology or the genomic location of markers, i.e genic vs. intergenic in eucalypts for growth and wood traits (Tan et al. 2017). Similarly, there were no noticeable differences between statistical methodologies used in maritime pine when comparing GBLUP and Bayesian methods (Isik et al. 2016). Depending on the importance of LD in contributing to accuracy of genomic predicted breeding values, Bayesian models may perform better than GBLUP for which the decay of the prediction accuracy tends to be larger, especially when the training data set is relatively small (Habier et al. 2010).

Forest tree breeding programmes are generally in the very early stages compared to animal and crop breeding programmes with faster generation turnover (Grattapaglia and Kirst 2008; Isik 2014). This causes genetic parameter estimates to be less accurate, as it is not always possible to take into account complete pedigrees, identify specific genetic groups or consider data since the selection began. Our analysis found that a marker-based predictions improved the accuracy of genetic parameter estimates, and also resulted in higher predictive accuracies in cross-validation evaluations than pedigreed-based breeding values. The likely source of this improvement is the utilization of all the available information in the populations through a complete pairwise marker-based relationship matrix accounting for realised genetic relationships between individuals (Zapata-Valenzuela et al. 2013). This, in conjunction with the faster progress in genetic improvement and delivery, are the major benefits to the implementation of genomics in forest tree breeding (Grattapaglia and Resende 2011).

In our study, the genomic information was available only for those individuals in the progeny test but not for their parents. Therefore, further improvement of genetic parameters through pedigree reconstruction cannot be achieved. Pedigree reconstruction is able to recover unknown relationships and correct inconsistencies in documented pedigrees (Doerksen and Herbinger 2010; El-Kassaby et al. 2011; Telfer et al. 2015; Vidal et al. 2015; Tan et al. 2018). More accurate genetic relationship information would increase the precision of genetic parameter estimations, and can be further explored through dense marker arrays that capture Mendelian sampling terms through the construction of a marker-based relationship matrix (Habier et al. 2007; Hayes et al. 2009; VanRaden 2008). In *Eucalyptus* hybrids, as a result of pedigree inconsistencies, genomic predictions outperformed pedigree-based predictions, which were largely underestimated (Tan et al. 2017). Sib-ship reconstruction was previously applied to the population used in this study by Klápště et al. (2017), who estimated the proportion of selfs being 4% in the population, with DBH and growth strain being the traits affected by inbreeding depression. Implementing sib-ship reconstruction increased genetic parameters and breeding value accuracies for the traits under inbreeding depression, and was consequently regarded as a useful tool to cull inbred individuals or selfs from the breeding population (Klápště et al. 2017). However, sib-ship reconstruction is not able to recover all classes of relatedness present in advanced breeding populations, and rather parentage reconstruction should be used when possible (Klápště et al. 2017).

Exceptionally low heritability for DBH and tree height in the current study is probably reflecting the composition of the training data set that is a selected subsample of the population. This resulted in a reduced genetic variance where the GBLUP implementation did not achieve any improvement in heritability estimates for those traits either. Between seed orchards, the marker-based predictions showed generally lower heritability estimates in Tinkers compared with Waiouru. This is interpreted as being a consequence of a higher selection intensity applied in the Tinkers population compared with Waiouru, which resulted in partial fixation of the genetic variance. The opposite overall trend for heritability estimates between the seed orchards was seen in the pedigree-based predictions. This difference can be explained by pedigree-based analysis overestimating additive genetic variance when a reference population is small. The across seed orchard estimates for heritability and breeding values accuracy converted to intermediate values between the within seed orchard estimates. Surprisingly, a larger sample size did not result in a higher accuracy of genetic parameters, which is attributed to be a consequence of merging two populations with different selection histories and smaller relatedness (Habier et al. 2013). The comparison of LD decay showed a strong difference between populations, which probably resulted in lack of marker effects transferability between merged populations.

Cross-validation was performed at an individual level to dissect the effects of genetic relationships, co-segregation of alleles, and LD between markers and QTL, three factors that genomic prediction is based on (Habier et al. 2013). The cross-validation captured all of the effects and showed a higher predictive accuracy in the Tinkers seed orchard compared to the Waiouru. This result was somewhat contrary to the estimated heritability and the accuracy estimates. The higher predictive accuracy in the Tinkers population can be explained by lower effective population size and larger haploblocks, which are built in populations created under higher selection intensity (supported by slower LD decay), and thus the whole genetic complex can be efficiently captured even by a sparse marker array (Ødegård and Meuwissen 2014). Transferability of this kind of prediction model is highly reduced, and can be seen in the cross-validation between seed orchards. As a result, when training the Waiouru seed orchard, a slightly higher predictive accuracy was found. However, the effect is limited due to lack of connectivity when using only one seed orchard as a training population to predict the other. The across population cross-validation again produced intermediate predictive accuracies between the seed orchards, but did not improve the estimates compared to the Tinkers population, in spite of an increase in training population size. Cross-validation performed in the same generation may not be ideal to estimate predictive accuracy for forward selection in the future breeding (Isik et al. 2016), whereas cross-validation over generations would likely result comparably better predictions (Isik et al. 2016). The accuracy of GEBVs varies depending on the training population size as well as the degree of genetic relationship between the training and validation population (e.g. Habier et al. 2010; Isik et al. 2016; Durán et al. 2017), which is recommended to be as high as possible. The relatedness is also a driver for building stronger LD through larger haploblocks and longer independent chromosomal segments, since the effective number of chromosomal segments is the outcome of the effective population size and length of the genome (Hayes et al. 2009). LD is the main contributor to the accuracy of GEBVs that is persistent over generations, therefore in case of the existence of considerable LD, a requirement for updating the phenotypes across generations is not so high (Habier et al. 2010). In spite of this, relying only on the accuracy originating from LD would result in smaller genetic gains than when the accuracy is based on both LD and relatedness between training and selection populations (Habier et al. 2007). Generally, it is highly recommended to capture a large proportion of the genetic variability in training populations in order to build robust genomic prediction models, making it important to keep a broad range of genetic material in training populations. Increasing the training population size does not only improve accuracy through higher relatedness but also through the increasing LD along the larger training population size (Habier et al. 2010). In genomics-based breeding programmes, the breeding archive should be established independently of the production seed orchards due to different requirements on genetic diversity vs. genetic gain trade-offs to utilize genomics at maximum efficiency (Grattapaglia and Resende 2011).

Genotype by environment interaction (GxE) plays a significant role in forestry tree breeding (Li et al. 2017), and is an important factor affecting transferability of prediction models. GxE would decrease the accuracy of genomic predicted breeding values as is the case also for pedigree-based estimations when genotypes are not stable across environments (Zapata-Valenzuela et al. 2013). GxE is a population and trait specific source of variation in forest tree breeding where both unstable genomic predictions (Resende et al. 2012b; Resende et al. 2012; Beaulieu et al. 2014a) as well as stable genomic predictions across environments have been reported (Lenz et al. 2017).

Estimated absolute genetic gains based on GBLUP breeding values per generation indicate that genomic selection would significantly improve the efficiency of selection for solid wood properties. The major benefit of genomic selection in accelerating the rate of genetic improvement would be derived from the ability to shorten the generation interval through the very early selection at the seedling stage (Resende et al. 2012, Beaulieu et al. 2014b). Improvement in selection efficiency was estimated at 50% in eucalypts if the breeding cycle was halved (Resende et al. 2012), and at 53–112% in loblolly pine (Resende et al. 2012b). Between 65 and 110% genetic gains were predicted for wood and growth traits in white spruce when relatedness between training and prediction data set was high, but lower as the relatedness decreased (Beaulieu et al. 2014b).

The potential to make faster selections by skipping progeny testing should be pursued in this *E. nitens* breeding population. A question remains as to how well prediction models perform after several breeding cycles (Resende et al. 2012), and how often the prediction models must be updated with new infusions of phenotypic data. Another aspect for consideration when using genomic prediction models are age-age correlations, since transferability of genomic models may be possible only when the selections are made at the same ages (Resende et al. 2012b). Additional genotyping from other progeny testing sites in the current population is recommended to ensure genomic prediction models are stable across sites. Further research on implications of genomic selection in this *E. nitens* breeding population is required to find the best possible methodology, including additional data infusions of wider breeding population that will reflect better the future selection population than in the current study. We expect further data infusions to result in considerably higher predictive accuracy of genomic breeding values compared to pedigree-based methods. Increasing the training population size as well as applying different statistical methods that can account efficiently for the accuracy due to LD may give further confidence in implementing GEBVs in the breeding programme (Habier et al. 2010). The benefits of genomic selection per unit of time for tree growers will be considerable, and therefore cost-effective ways to apply genomics in the operational breeding should also be the focus of future research.

## Conclusions

This study showed that a significant improvement in breeding value accuracy and genetic gains for selection of wood properties in *E. nitens* was possible by implementing genomic marker-based prediction compared to pedigree-based prediction. The greatest improvement in genetic parameters was obtained for tangential air-dry wood shrinkage and growth strain, which are the key traits in selection for solid wood production in eucalypts. Wood shrinkage traits had moderate heritabilities, which mainly increased further with genomic prediction.

Results from cross-validation analysis implied that further infusions of additional seed-orchard material into the training data would be useful to increase the efficiency of genomics in the selection, regarding breeding value accuracy and predictive accuracy. Further analysis, including more progeny trial sites to investigate the transferability of these models across generations and environments is recommended.

### Data availability

Data available from Dryad: 10.5061/dryad.pf58510.
