# Correction to: Efficiency of genomic prediction across two *Eucalyptus nitens* seed orchards with different selection histories

**DOI:** 10.1038/s41437-018-0159-x

**Published:** 2018-10-22

**Authors:** Mari Suontama, Jaroslav Klápště, Emily Telfer, Natalie Graham, Toby Stovold, Charlie Low, Russell McKinley, Heidi Dungey

**Affiliations:** Scion (The New Zealand Forest Research Institute Ltd.), 49 Sala Street, Rotorua, 3046 New Zealand

Correction to: *Heredity*; 10.1038/s41437-018-0119-5; Published online: 6 July 2018

This article was originally published under standard licence, but has now been made available under a [CC BY 4.0] license. The PDF and HTML versions of the paper have been modified accordingly.

